# Genetic spectrum of familial hypercholesterolemia and correlations with clinical expression: Implications for diagnosis improvement

**DOI:** 10.1111/cge.14036

**Published:** 2021-08-03

**Authors:** Maria Donata Di Taranto, Carola Giacobbe, Daniela Palma, Gabriella Iannuzzo, Marco Gentile, Ilenia Calcaterra, Ornella Guardamagna, Renata Auricchio, Matteo Nicola Dario Di Minno, Giuliana Fortunato

**Affiliations:** ^1^ Dipartimento di Medicina Molecolare e Biotecnologie Mediche Università degli Studi di Napoli Federico II, CEINGE Biotecnologie Avanzate s.c. a r.l. Naples Italy; ^2^ Dipartimento di Medicina Clinica e Chirurgia Università degli Studi di Napoli Federico II Naples Italy; ^3^ Dipartimento di Scienze della Sanità Pubblica e Pediatriche Università degli Studi di Torino Turin Italy; ^4^ Dipartimento di Scienze Mediche Traslazionali Università degli Studi di Napoli Federico II Naples Italy

**Keywords:** genotype–phenotype correlation, homozygous familial hypercholesterolemia, null variant, pathogenic variant, pediatric FH, variant cluster

## Abstract

Familial hypercholesterolemia (FH) is the most common genetic disease caused by variants in *LDLR*, *APOB*, *PCSK9* genes; it is characterized by high levels of LDL‐cholesterol and premature cardiovascular disease. We aim to perform a retrospective analysis of a genetically screened population (528 unrelated patients—342 adults and 186 children) to evaluate the biochemical and clinical correlations with the different genetic statuses. Genetic screening was performed by traditional sequencing and some patients were re‐analyzed by next‐generation‐sequencing. Pathogenic variants, mainly missense in the *LDLR* gene, were identified in 402/528 patients (76.1%), including 4 homozygotes, 17 compound heterozygotes and 1 double heterozygotes. A gradual increase of LDL‐cholesterol was observed from patients without pathogenic variants to patients with a defective variant, to patients with a null variant and to patients with two variants. Six variants accounted for 51% of patients; a large variability of LDL‐cholesterol was observed among patients carrying the same variant. The frequency of pathogenic variants gradually increased from unlikely FH to definite FH, according to the Dutch Lipid Clinic Network criteria. Genetic diagnosis can help prognostic evaluation of FH patients, discriminating between the different genetic statuses or variant types. Clinical suspicion of FH should be considered even if few symptoms are present or if LDL‐cholesterol is only mildly increased.

## Graphical abstract

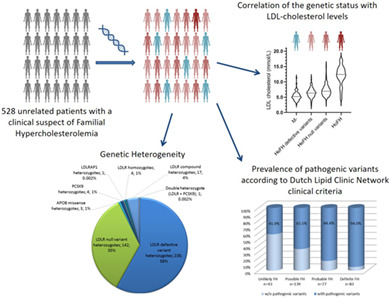

## 1 INTRODUCTION

Familial hypercholesterolemia (FH [MIM]: 143890, 144010 and 603776) is the most common genetic disease, characterized by high plasma levels of low‐density lipoprotein cholesterol (LDL‐c) and early cardiovascular disease.^1^ Despite early identification of FH patients being crucial to prevent cardiovascular events, FH remains greatly underdiagnosed.^2^ FH is inherited as autosomal dominant condition and the frequency of heterozygous FH (HeFH) is ~1:200.^2^, ^3^ The FH is mainly due to the presence of loss‐of‐function variants in low density lipoprotein receptor (*LDLR*) gene (MIM: 606945), while loss‐of‐function variants in apolipoprotein B (*APOB*) gene (MIM: 107730) and gain‐of‐function variants in proprotein convertase subtilisin/kexin type 9 (*PCSK9*) gene (MIM: 607786) are less frequently.^4^ A pathogenic variant in apolipoprotein E (*APOE*) gene (MIM: 107741) is also described like causative of FH.^5^ Pathogenic variants in low density lipoprotein receptor adaptor protein 1 (*LDLRAP1*) gene (MIM: 605747) are causative of a recessive form of hypercholesterolemia (ARH).^6^ In addition to HeFH also a homozygous FH (HoFH) form has been described; this latter is caused by the presence of pathogenic variants on two alleles of candidate genes (homozygosis, compound heterozygosis or double heterozygosis) and recent data suggest a prevalence of ~1:300.000.^7^ The HoFH form is more severe than HeFH, with clinical manifestations of atherosclerosis reported in early childhood. To date, the Dutch Lipid Clinic Network (DLCN) diagnostic criteria^2^ and Simon Broome Register diagnostic criteria^8^ are largely used to clinical diagnose FH and consider the presence of a pathogenic variant as one of the major determinants in definite diagnosis.

Previous reports about large Italian FH population have been published. The most recent one analyzed the genetic architecture of FH in patients collected by lipid clinics across the whole nation but did not performed a genotype–phenotype correlation.^9^ This aspect was investigated by another Italian study revealing differences between *LDLR* variants leading to receptor defective or receptor negative proteins.^10^ However, these studies did not perform a separate analysis and a comparison between adults and children.

The detection of a pathogenic variant in one of candidate genes is a cardiovascular risk factor independently from the LDL‐c levels.^11^ The variant identified in a patient can be searched in the patient relatives (cascade screening) improving the identification and early treatment of additional FH patients.^12^ However, in about 20%–30% of patients with a clinical suspicion of FH no pathogenic variants are identified^2^; a few such patients could be carriers of pathogenic variants which caused other rare diseases whose phenotype overlaps with FH such as Sitosterolaemia (MIM: 210250 and 618 666) or Lysosomal Acid Lipase Deficiency (MIM: 278000) (FH phenocopies).^4^, ^13^ A polygenic base has been hypothesized considering the accumulation of common small‐effect LDL‐c‐raising alleles,^14^ although the diagnostic power of the calculated risk score are not useful for FH diagnosis.^15^

Here we report the genetic spectrum emerging from the retrospective analysis of an Italian population genetically screened in the last 11 years highlighting the complexity of FH genetics. We also analyzed the genotype–phenotype correlations and the clinical features associated with the different variant types.

## 2 MATERIALS AND METHODS

### 2.1 Patients

This retrospective study is based on a population of adult (>16 years) and pediatric patients (≤16 years) with a clinical suspicion of FH genetically analyzed between 2008 and 2019 by the Dipartimento di Medicina Molecolare e Biotecnologie Mediche of the Università degli Studi di Napoli Federico II, and CEINGE. Most of patients were recruited at the Dipartimento di Medicina Clinica e Chirurgia (adult lipid clinic) and Dipartimento di Scienze Mediche Traslazionali (pediatric lipid clinic) of Università degli Studi di Napoli Federico II; at the Dipartimento di Scienze della Sanità Pubblica e Pediatriche of Università degli Studi di Torino. This study also included patients addressed to genetic screening by unknown physicians; for these patients several clinical and familial data were missing.

Recruitment criteria are LDL‐cholesterol (LDL‐c) > 4 mmol/L in adults or LDL‐c > 3.4 mmol/L in children together with a family history of hypercholesterolemia (LDL‐c > 4.9 mmol/L or total cholesterol >6.5 mmol/L) and/or of premature coronary artery disease (<55 years in men or <60 years in women). Patients with LDL‐c levels lower than these thresholds were included if a typical dominant transmission of hypercholesterolemia was reported in the family.

The study included one patient per family, namely 528 patients (342 adults and 186 children), which characteristics are reported in Table S1.

A written informed consent was collected for each patient. The study was performed according to the current version of the Helsinki Declaration and then approved by the Ethical Committee of the “Università degli Studi di Napoli Federico II” (Number 262/17, November 29, 2017).

### 2.2 Clinical assessment and biochemical evaluation

Patients were questioned about personal and family history of hypercholesterolemia and cardiovascular diseases; presence of tendon xanthomas, corneal arcus and carotid plaque were also verified. Tendon xanthomas were considered present if at the inspection and palpation of Achilles tendons, tendon at the dorsum of hands, elbows and knees a diffuse enlargement or nodules are present. Other collected data are: smoking habits, presence of other diseases (such as hypertension, diabetes, thyroid dysfunction). Body mass index (BMI) was calculated as weight (kg)/height^2^ (m^2^).

Total cholesterol, HDL‐cholesterol (HDL‐c) and triglycerides reported in this study have been evaluated in absence of lipid‐lowering therapy and were measured by standard enzymatic methods, whereas LDL‐c was calculated by the Friedewald formula. In case of patients on therapy at the first observation, the pre‐therapy LDL‐c was calculated according to a formula previously.^16^ The non‐HDL‐cholesterol (non‐HDL‐c) and the LDL/HDL ratio were also calculated.

Clinical diagnostic criteria for FH diagnosis were applied: Dutch Lipid Clinic Network (DLCN) Score was calculated for adult patients and interpreted according to Nordestgaard et al.^2^; Simon Broome diagnostic criteria were considered for all patients.^8^ Because some clinical and familial information were difficult to retrieve for several patients, 43 patients were classified as “Unlikely FH” and 267 as “No FH” according to DLCN and Simon Broome, respectively.

### 2.3 Genetic screening

Genetic screening included the sequence analysis of all *LDLR* exons together with the intron‐exon junctions as previously reported.^17^ If no pathogenic variants were detected, large rearrangements in LDLR were searched by Multiplex Ligation‐dependent Probe Amplification. When no pathogenic variants in *LDLR* were detected, the screening of *PCSK9* was performed by sequence analysis of all exons together with the intron‐exon junctions, while *APOB* sequence analysis was limited to the region coding for the LDLR binding region (a portion of exon 26 and the whole exon 29 with the intron‐exon junctions as described in Rubba et al.^18^). Finally, the screening was extended to all *LDLRAP1* exons together with the intron‐exon junctions, if no pathogenic variants in *LDLR*, *APOB* and *PCSK9* genes were found. For homozygotes/compound heterozygotes, the variant presence was ascertained in both parents, allowing to confirm that the two variants were present on the two different alleles. This was not performed for the double heterozygote, but as the two variants are present in two different genes on two different chromosomes, it was unnecessary to perform further analyses to establish the patient genotype.

In a subgroup of 49 patients for which the DNA sample was available and of good quality, we searched for other possible pathogenic variants by next generation sequencing (NGS) using a panel comprising promoter, all exons and exon‐intron junctions of *LDLR*, *APOB*, *PCSK9*, *LDLRAP1*, *APOE* and *STAP1* genes (Devyser FH kit, Devyser, Sweden). A 200‐bp amplicons amplification was performed in a single tube starting from genomic DNA (2 ng/μl) quantified by Qubit® 2.0 Fluorometer 8 (Life Technologies). Sequencing was performed by Kit v2 Micro on a MiSeq platform (Illumina) with paired‐end reads (2 × 150 base pairs). Amplicon Suite software (SmartSeq) was used to analyze FastQ files and to perform the functional annotation of identified variants. This kit also allows to detect copy number variant of *LDLR* gene.

All variants were reported according to the Human Genome Variation Society nomenclature using these reference sequence: *LDLR* (LRG_274t1; NM_000527.4; NP_000518.1), *APOB* (NM_000384.3; NP_000375.3), *PCSK9* (LRG_275t1; NM_174936.3; NP_777596.2); *LDLRAP1* (LRG_276t1; NM_015627.2; NP_056442.2).

### 2.4 Pathogenicity evaluation

For all rare variants the pathogenic evaluation was performed according to the American College of Medical Genetics and Genomics (ACMG) Guidelines^19^ and recent FH‐specific suggestions.^20^

To verify the minor allele frequency (MAF), the variants identified during screening were searched in Varsome database^21^ and then in the Genome Aggregation Database (GnomAD—https://gnomad.broadinstitute.org/); variants with MAF less than 1% were considered rare. These latter were checked against pathogenic variants databases: the Leiden Open Variation Database (LOVD—https://databases.lovd.nl/shared/genes/LDLR) and the Human Gene Mutation Database (HGMD).

Missense, deletion/insertion without frameshift and promoter variants in the *LDLR* gene were considered defective variants, whereas nonsense, splicing, deletion/insertion leading to frameshift and large rearrangements were defined null variants as reported in ACMG guidelines.^19^ Missense variants in *APOB* and *PCSK9* genes were considered defective because the protein alteration did not lead to a complete loss of LDL‐LDLR binding and uptake.

### 2.5 Statistical analysis

Continuous variables are reported as median and interquartile range because at the normality test (Kolmogorov–Smirnov) their distribution resulted not parametric. Categorical variables were expressed and absolute number and percentage. Comparisons between two groups were performed by Mann–Whitney test for continuous variables and Fisher exact test for categorical variables.

Analysis of receiver operating characteristic (ROC) curves was performed by MedCalc and the given significances are referred on the difference between the area under the curve (AUC) and the area under the bisector. The farthest point from the bisector was considered the best cut‐off.

Multiple comparisons among genetic statuses and related plots have been performed by Prism 8 (GraphPad). All other analyses have been performed by SPSS (IBM). A *p* < 0.05 was considered significant.

## 3 RESULTS

### 3.1 Genetic spectrum of FH

Pathogenic variants were identified in 402/528 unrelated patients (76.1%), 380 of which are HeFH (94.5%) and 22 are HoFH (5.5%). Notably, among all patients we identified 25 HoFH, because 3 siblings were present in the study. Dividing the patients according to age, we found pathogenic variants in 252/342 adult patients (73.7%) and in 150/186 pediatric patients (80.6%); this difference was not significant at fisher exact test. Overall, most of patients carried a pathogenic variant in the *LDLR* gene (394/402 patients; 98.0%), confirming that this gene is the main responsible of FH in our population.

Among HeFH patients, 372 carried pathogenic variants in *LDLR*, 3 in *APOB*, 4 in *PCSK9* and 1 in *LDLRAP1* genes. All variants in *APOB*, *PCSK9* and *LDLRAP1* are missense variants, whereas as to *LDLR*, 230 carried defective variants (223 missense) and 142 patients carried null variants. Among HoFH patients, 4 patients were true homozygotes, 17 were compound heterozygotes and 1 was double heterozygote. Most of these have been recently described^22^ and 2 were later identified, including a double heterozygote (6.35 mmol/L of LDL‐c) for the deletion of exons 11–18 ‐ c.(1586 + 1_1587 − 1)_(*450_?)del—of *LDLR* gene and the *PCSK9* variant p.(Ser636Arg) also identified at heterozygous status. Table S2 reports the genetic status of all 22 unrelated HoFH. As 21 unrelated HoFH patients originate from a single Italian region (Campania), lived by about 6 000 000 inhabitants, we were able to estimate the prevalence of HoFH in this region as 1:286000.

**FIGURE 1:**
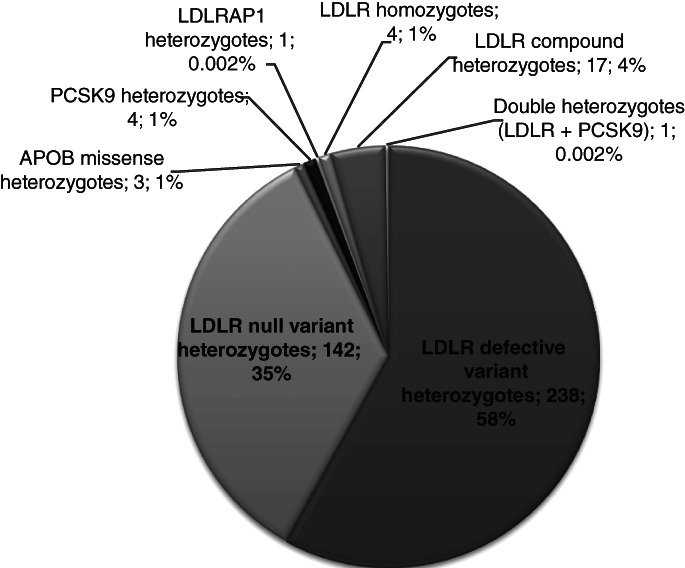
Pie graph reporting the different genetic statuses observed in the studied population. The number of patients with each genetic status is indicated with the first number together with the percentage relative to the patients with pathogenic variants (*n* = 402)

A report of the frequencies of the different genetic statuses is reported in Figure 1, whereas a plot of all different variants found at heterozygous status is reported in Figure S1. Interestingly, the 6 most frequent variants account for 36.7% of all unrelated patients (194/528 patients), namely 51% (194/380) of HeFH patients (Figure S2). These variants are all present in the *LDLR* gene: c.2312‐3C>A, c.1586+1G>A, p.(Gly592Glu), p.(Gly549Asp), p.(Val523Met) and p.(Cys379Arg). On the other hand, several variants have been found only in single patients, according to the high genetic heterogeneity typical of FH. Table 1 reports all the pathogenic and likely pathogenic variants identified in HeFH patients. Considering both HeFH and HoFH patients we identified 107 different pathogenic variants: 99 in *LDLR* gene, 3 in *APOB* gene, 4 in *PCSK9* gene and 1 in *LDLRAP1* gene.

**TABLE 1:** Frequency of pathogenic and likely pathogenic variants identified in HeFH patients

Gene	Region	Nucleotide	Protein	Defective/null variant	Number of HeFH patients	Variant ID and MAF in GnomAD
*LDLR*	exon 2	LRG_274t1:c.81C>G	p.(Cys27Trp)	Defective	1	rs2228671; *G* = 0.00001595
*LDLR*	exon 2	LRG_274t1:c.97C>T	p.(Gln33*)	Null	1	rs121908024; *T* = 0.000007963
*LDLR*	exon 2	LRG_274t1:c.102C>G	p.(Cys34Trp)	Defective	1	n.a.; n.a.
*LDLR*	exon 2	LRG_274t1:c.116_117delGCinsAA	p.(Cys39*)	Null	4	rs879254412; n.a.
*LDLR*	exon 3	LRG_274t1:c.233delG	p.(Arg78Leufs*128)	Null	2	rs1057516129; n.a.
*LDLR*	exon 3	LRG_274t1:c.304C>T	p.(Gln102*)	Null	4	rs563390335; *T* = 0.000003977
*LDLR*	intron 3	LRG_274t1:c.313+1G>A	p.(Leu64_Pro105delinsSer)	Null	2	rs112029328; *A* = 0.00002784
*LDLR*	exon 4	LRG_274t1:c.337G>T	p.(Glu113*)	Null	1	rs769383881; n.a.
*LDLR*	exon 4	LRG_274t1:c.352G>T	p.(Asp118Tyr)	Defective	5	rs730882080; *T* = 0.00001196
*LDLR*	exon 4	LRG_274t1:c.367 T>C	p.(Ser123Pro)	Defective	2	rs879254495; n.a.
*LDLR*	exon 4	LRG_274t1:c.369_370delTC	p.(Arg124Alafs*5)	Null	1	rs879254496; n.a.
*LDLR*	exon 4	LRG_274t1:c.418G>T	p.(Glu140*)	Null	3	rs748944640; *T* = 0.000007963
*LDLR*	exon 4	LRG_274t1:c.424_430delTCCTGCC	p.(Ser142Argfs*62)	Null	1	rs879254521; n.a.
*LDLR*	exon 4	LRG_274t1:c.440C>T	p.(Thr147Ile)	Defective	3	rs879254524; n.a.
*LDLR*	exon 4	LRG_274t1:c.465C>A	p.(Cys155*)	Null	8	rs766094434; *A* = 0.000003980
*LDLR*	exon 4	LRG_274t1:c.514G>A	p.(Asp172Asn)	Defective	1	rs879254554; *A* = 0.00003185
*LDLR*	exon 4	LRG_274t1:c.542C>G	p.(Pro181Arg)	Defective	2	rs557344672; *G* = 0.00002388
*LDLR*	exon 4	LRG_274t1:c.551G>A	p.(Cys184Tyr)	Defective	1	rs121908039; *A* = 0.00009554
*LDLR*	exon 4	LRG_274t1:c.662A>G	p.(Asp221Gly)	Defective	5	rs373822756; *G* = 0.00005204
*LDLR*	exon 4	LRG_274t1:c.664_681dup	p.(Cys222_Asp227dup)	Defective	1	rs387906306; dup = 0.000004000
*LDLR*	exon 4	LRG_274t1:c.666C>A	p.(Cys222*)	Null	1	rs756613387; *A* = 0.000004005
*LDLR*	exon 4	LRG_274t1:c.671A>G	p.(Asp224Gly)	Defective	1	rs879254630; n.a.
*LDLR*	exon 4	LRG_274t1:c.673_681dup	p.(Lys225_Asp227dup)	Defective	1	rs1555803425; n.a.
*LDLR*	exon 4	LRG_274t1:c.680_692delACGAGGAAAACTG	p.(Asp227Alafs*34)	Null	1	rs1057519660; n.a.
*LDLR*	exon 4	LRG_274t1:c.681C>G	p.(Asp227Glu)	Defective	1	rs121908028; *G* = 0.000008060
*LDLR*	exon 4	LRG_274t1:c.648dupT	p.(Asp217*)	Null	1	n.a.; n.a.
*LDLR*	intron 4	LRG_274t1:c.694+1G>C	p.(?)	Null	1	rs879254646; n.a.
*LDLR*	exon 5	LRG_274t1:c.718G>A	p.(Glu240Lys)	Defective	1	rs768563000; *A* = 0.00003181
*LDLR*	exon 5	LRG_274t1:c.761A>C	p.(Gln254Pro)	Defective	2	rs879254667; n.a.
*LDLR*	exon 5	LRG_274t1:c.788A>G	p.(Asp263Gly)	Defective	1	rs141681167; n.a.
*LDLR*	exon 5	LRG_274t1:c.808 T>C	p.(Cys270Arg)	Defective	1	rs879254682; n.a.
*LDLR*	exon 6	LRG_274t1:c.859G>T	p.(Gly287Cys)	Defective	1	rs375495026; n.a.
*LDLR*	exon 6	LRG_274t1:c.922G>A	p.(Glu308Lys)	Defective	1	rs879254721; n.a.
*LDLR*	exon 7	LRG_274t1:c.953G>T	p.(Cys318Phe)	Defective	10	rs879254739; n.a.
*LDLR*	exon 7	LRG_274t1:c.974G>A	p.(Cys325Tyr)	Defective	3	rs879254746; n.a.
*LDLR*	exon 7	LRG_274t1:c.1003G>A	p.(Gly335Ser)	Defective	2	rs544453230; *A* = 0.00002833
*LDLR*	exon 7	LRG_274t1:c.1027G>A	p.(Gly343Ser)	Defective	1	rs730882096; *A* = 0.00002832
*LDLR*	exon 7	LRG_274t1:c.1056C>G	p.(Cys352Trp)	Defective	1	rs13306515; n.a.
*LDLR*	intron 7	LRG_274t1:c.1060+10G>A	p.(Asp354Glyfs*20)	Null	1	rs12710260; *A* = 0.00001200
*LDLR*	intron 7	LRG_274t1:c.1061‐1G>T	p.(?)	Null	1	n.a.; n.a.
*LDLR*	exon 8	LRG_274t1:c.1070_1071dupAG	p.(Cys358Serfs*13)	Null	1	n.a.; n.a.
*LDLR*	exon 8	LRG_274t1:c.1118G>A	p.(Gly373Asp)	Defective	1	rs879254797; *A* = 0.000003537
*LDLR*	exon 8	LRG_274t1:c.1120_1123dupGGCT	p.(Tyr375Trpfs*7)	Null	2	rs879254799; n.a.
*LDLR*	exon 8	LRG_274t1:c.1130G>T	p.(Cys377Phe)	Defective	2	rs879254801; n.a.
*LDLR*	exon 8	LRG_274t1:c.1135 T>C	p.(Cys379Arg)	Defective	22	rs879254803; *C* = 0.00003185
*LDLR*	exon 9	LRG_274t1:c.1187delG	p.(Gly396Alafs*17)	Null	1	rs1057519667; n.a.
*LDLR*	exon 9	LRG_274t1:c.1207_1209delTTC	p.(Phe403del)	Defective	1	n.a.; n.a.
*LDLR*	exon 9	LRG_274t1:c.1211C>T	p.(Thr404Ile)	Defective	2	rs879254835; n.a.
*LDLR*	exon 9	LRG_274t1:c.1215C>G	p.(Asn405Lys)	Defective	1	rs879254837; n.a.
*LDLR*	exon 9	LRG_274t1:c.1216C>A	p.(?)—splicing alteration	Null	1	rs121908043; n.a.
*LDLR*	exon 9	LRG_274t1:c.1247G>C	p.(Arg416Pro)	Defective	1	rs773658037; n.a.
*LDLR*	exon 9	LRG_274t1:c.1277 T>G	p.(Leu426Arg)	Defective	2	n.a.; n.a.
*LDLR*	exon 9	LRG_274t1:c.1295 T>C	p.(Leu432Pro)	Defective	3	rs879254855; n.a.
*LDLR*	exon 9	LRG_274t1:c.1331C>T	p.(Ser444Phe)	Defective	1	n.a.; n.a.
*LDLR*	exon 10	LRG_274t1:c.1414G>T	p.(Asp472Tyr)	Defective	1	rs730882102; *T* = 0.00005308
*LDLR*	exon 10	LRG_274t1:c.1415_1418dupACAT	p.(Gln474Hisfr*63)	Null	1	rs879254892; n.a.
*LDLR*	exon 10	LRG_274t1:c.1466A>G	p.(Tyr489Cys)	Defective	1	rs879254914; n.a.
*LDLR*	exon 10	LRG_274t1:c.1474G>A	p.(Asp492Asn)	Defective	5	rs373646964; *A* = 0.000007072
*LDLR*	exon 10	LRG_274t1:c.1478_1479delCT	p.(Ser493Cysfs*42)	Null	3	rs869025453; CT = 0.00003191
*LDLR*	exon 10	LRG_274t1:c.1558A>G	p.(Arg520Gly)	Defective	1	rs879254939; n.a.
*LDLR*	exon 10	LRG_274t1:c.1567G>A	p.(Val523Met)	Defective	26	rs28942080; *A* = 0.00001194
*LDLR*	exon 10	LRG_274t1:c.1574A>T	p.(Asp525Val)	Defective	1	rs879254943; n.a.
*LDLR*	intron 10	LRG_274t1:c.1586+1G>A	p.(Thr454_Gly529del) and p.(Gly529_Phe530ins22)	Null	29	rs755389753; *A* = 0.000003982
*LDLR*	exon 11	LRG_274t1:c.1646G>A	p.(Gly549Asp)	Defective	29	rs28941776; *A* = 0.00002386
*LDLR*	exon 11	LRG_274t1:c.1685G>A	p.(Trp562*)	Null	1	rs875989925; n.a.
*LDLR*	exon 11	LRG_274t1:c.1686G>T	p.(Trp562Cys)	Defective	1	n.a.; n.a.
*LDLR*	exon 11	LRG_274t1:c.1694G>T	p.(Gly565Val)	Defective	2	rs28942082; n.a.
*LDLR*	exon 11	LRG_274t1:c.1698_1704delinsGCCCAAT	p.(Ile566_Leu568delinsMetProAsn)	Defective	4	rs879254989; n.a.
*LDLR*	exon 12	LRG_274t1:c.1720C>T	p.(Arg574Cys)	Defective	1	rs185098634; *T* = 0.00003535
*LDLR*	exon 11	LRG_274t1:c.1775G>A	p.(Gly592Glu)	Defective	1	rs137929307; *A* = 0.00005656
*LDLR*	exon 12	LRG_274t1:c.1731G>C	p.(Trp577Cys)	Defective	1	rs875989928; n.a.
*LDLR*	exon 12	LRG_274t1:c.1735G>T	p.(Asp579Tyr)	Defective	1	rs875989929; n.a.
*LDLR*	exon 12	LRG_274t1:c.1739C>T	p.(Ser580Phe)	Defective	8	rs934496989; *T* = 0.000003976
*LDLR*	exon 12	LRG_274t1:c.1775G>A	p.(Gly592Glu)	Defective	48	rs137929307; *A* = 0.00005656
*LDLR*	exon 13	LRG_274t1:c.1898G>A	p.(Arg633His)	Defective	1	rs754536745; *A* = 0.00002121
*LDLR*	intron 13	LRG_274t1:c.1846c1G>A	p.([(Glu615fs*43, Leu570_Thr621del, Glu615fs*16)]	Null	3	rs879255051; *A* = 0.00003184
*LDLR*	exon 13	LRG_274t1:c.1943_1944delinsG	p.(Ser648Cysfs*17)	Null	1	n.a.; n.a.
*LDLR*	exon 14	LRG_274t1:c.2054C>T	p.(Pro685Leu)	Defective	9	rs28942084; *T* = 0.00003184
*LDLR*	exon 15	LRG_274t1:c.2215C>T	p.(Gln739*)	Null	1	rs370018159; n.a.
*LDLR*	intron 15	LRG_274t1:c.2311+1G>A	p.(Gln770_Ala771ins30) and p.(Lys730fs*16) and p.(Glu714_Gln770del)	Null	1	rs879255175; n.a.
*LDLR*	intron 15	LRG_274t1:c.2312‐3C>A	p.(Ala771_Ile796del)	Null	39	rs875989942; n.a.
*LDLR*	exon 16	LRG_274t1:c.2389G>A	p.(Val797Met)	Defective	4	rs750518671; *A* = 0.000007957
*LDLR*	del exon 13–14	LRG_274t1:c.(1845 + 1_1846 − 1)_(2140 + 1_2141 − 1)del	p.(Asp616Argfs*16)	Null	1	n.a.; n.a.
*LDLR*	del exon 11–12	LRG_274t1:c.(1586 + 1_1587 − 1)_(1845 + 1_1846‐1)del	p.(Phe530Thrfs*49)	Null	13	n.a.; n.a.
*LDLR*	del exon 13–15	LRG_274t1:c.(1845 + 1_1846 − 1)_(2311 + 1_2312‐1)del	p.(Asp616Leufs*17)	Null	7	n.a.; n.a.
*LDLR*	del exon 15–16	LRG_274t1:c.(2140 + 1_2141 − 1)_(2389 + 1_2390‐1)del	p.(Glu714_Ile796del)	Null	1	n.a.; n.a.
*LDLR*	del exon 2–12	LRG_274t1:c.(67 + 1_68 − 1)_(1845 + 1_1846‐1)del	p.(Val23Glyfs*29)	Null	1	n.a.; n.a.
*LDLR*	del exon 12	LRG_274t1:c.(1705 + 1_1706 − 1)_(1845 + 1_1846‐1)del	p.(?)	Null	1	n.a.; n.a.
*LDLR*	intron 17	LRG_274t1:c.2547+2T>G	p.(?)	Null	1	n.a.; n.a.
*APOB*	exon 26	NM_000384.3:c.10672C>T	p.(Arg3558Cys)	Defective	1	rs12713559; *A* = 0.0003577
*APOB*	exon 26	NM_000384.3:c.10679A>G	p.(Tyr3560Cys)	Defective	1	rs745721296; *C* = 0.00001195
*APOB*	exon 26	NM_000384.3:c.10708C>T	p.(His3570Tyr)	Defective	1	rs201736972; *A* = 0.0001311
*PCSK9*	exon 1	LRG_275t1:c.103G>T	p.(Asp35Tyr)	GOF/defective LDLR activity	1	rs764603059; *T* = 0.00001077
*PCSK9*	exon 7	LRG_275t1:c.1069C>T	p.(Arg357Cys)	GOF/defective LDLR activity	1	rs148562777; *T* = 0.0001450
*PCSK9*	exon 9	LRG_275t1:c.1496G>A	p.(Arg499His)	GOF/defective LDLR activity	1	rs143394031; *A* = 0.00001819
*PCSK9*	exon 12	LRG_275t1:c.1906A>C	p.(Ser636Arg)	GOF/defective LDLR activity	1	n.a.; n.a.
*LDLRAP1*	exon 6	LRG_276t1:c.605C>A	p.(Ser202Tyr)	Defective	1	rs121908326; *A* = 0.001188

Abbreviations: GOF, gain of function variant; MAF, minor allele frequency; n.a., not available.

Additional rare variants of uncertain significance (VUS), that is, variants which the pathogenic evaluation does not allowed to establish a clear pathogenic or benign role, have been identified. These VUS were detected at higher frequency in patients without pathogenic variants 32/126 (25.4%) than in patients with pathogenic variants 18/402 (4.5%), *p* < 0.00001 at Fisher test.

As a pathogenicity criterion is based on the previous identification of variants in additional FH patients, we report the VUS in Table S3 in order to facilitate future studies on FH genetics.

We further sequenced a subgroup of 49 patients (38 adults and 11 children) with different genetic status: 29 without pathogenic variants, 17 HeFH for variants in *LDLR*, 1 HeFH for a single variant in *LDLRAP1* gene, 2 compound heterozygotes for *LDLR* variants and 1 double heterozygote for variants in *LDLR* and *PCSK9* genes. All previously identified variants were confirmed and no additional rare variants (pathogenic, VUS or benign) were identified in these patients and consequently no changes in the previous genetic diagnosis were present.

### 3.2 Genotype–phenotype correlation analysis

**FIGURE 2:**
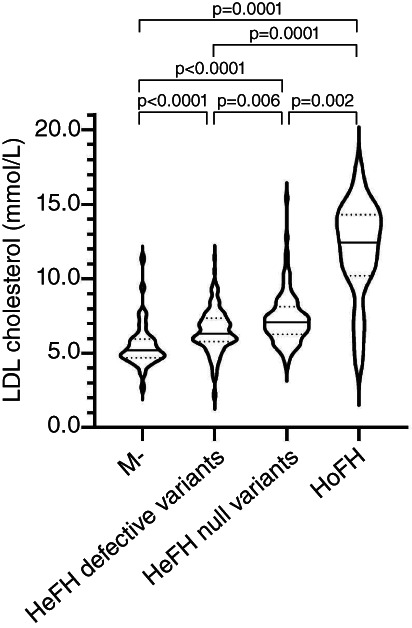
Violin plot representing low‐density lipoprotein (LDL)‐cholesterol values observed in patients with different genetic statuses. The violin shape represents the smoothed frequency distribution of the LDL‐cholesterol values expressed in mmol/L. The continuous horizontal line within each value represents the distribution median, whereas the dashed lines represent the first and the third quartile of value distribution in each group. Statistical significances corrected for multiple comparisons obtained by Dunn's test are reported. HeFH defective, heterozygous patients with defective variants; HeFH null, heterozygous patients with null variants; HoFH, homozygous patients; M, patients without pathogenic variants

We compared the untreated LDL‐c levels of patients with different genetic statuses, further distinguishing HeFH in patients with a defective or a null variant. The violin plot reported in Figure 2 shows the gradual increase of LDL‐c observed from patients without pathogenic variants to patients with a defective variant, to patients with a null variant and to HoFH patients. This plot also highlights the great variability of LDL‐c levels in each group. As these groups contains both pediatric and adult patients, we repeated the analysis further dividing patients for age (Figure S3). By this analysis, we observed no differences of LDL‐c between children carrying a defective or a null variant or between HeFH and HoFH. Only the differences between patients without a pathogenic variant and the other three groups were statistically significant.

Analyzing only the heterozygous patients with a missense variant, we verified that patients with the variant in *LDLR* gene (*n* = 223) showed a worse phenotype than patients with a variant in *APOB* or *PCSK9* genes (*n* = 7), showing higher LDL‐c values (6.53 ± 1.56 mmol/L vs. 5.26 ± 1.74 mmol/L, respectively; *p* = 0.036) and higher values of LDL/HDL ratio (5.12 ± 1.77 vs. 3.31 ± 1.18, respectively; *p* = 0.008).

**FIGURE 3:**
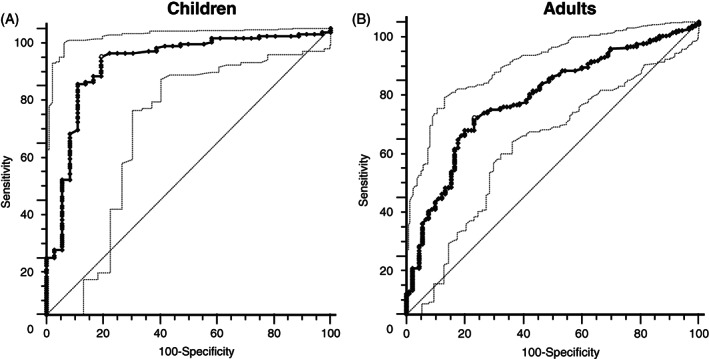
Receiver operating characteristic (ROC) curves evaluating the ability of low‐density lipoprotein (LDL)‐cholesterol values to distinguish between patients with and without pathogenic variants. The ROC curve is indicated with bold line and open circles represent the best criterion points. Light line indicates the 95% confidence interval (CI). Dashed line indicates the bisector. AUC, area under the curve

We also evaluated the ability of lipid parameters to distinguish between patients with and without pathogenic variants through ROC curves performed separating pediatric and adult patients. As expected, results highlighted that the best parameter allowing to distinguish patients with and without pathogenic variants is the LDL‐c (Figures 3 and S4) with an AUC of 0.878 (*p* < 0.0001) for children and 0.739 (*p* < 0.0001) for adults. Notably, for all parameters the ROC curves relative to pediatric patients showed a higher AUC respect to those relative to adult patients. The best cut‐off of LDL‐c, defined as the farthest point from the bisector were 4.9 mmol/L (188 mg/dl—sensitivity 90.0% and specificity 80.6%) in children and 6.24 mmol/L (241 mg/dl—sensitivity 67.1% and specificity 76.7%) in adults. On the other hand, to reach 100% of sensitivity the values of 2.87 mmol/L (111 mg/dl) and 2.07 mmol/L (80 mg/dl) should be considered in children and adults, respectively. Notably, the non‐HDL cholesterol showed a discriminating power similar to that of LDL‐c. We also observed that an LDL/HDL value higher than 5.21 allows to obtain 100% of specificity in identifying pediatric patients with pathogenic variants.

**FIGURE 4:**
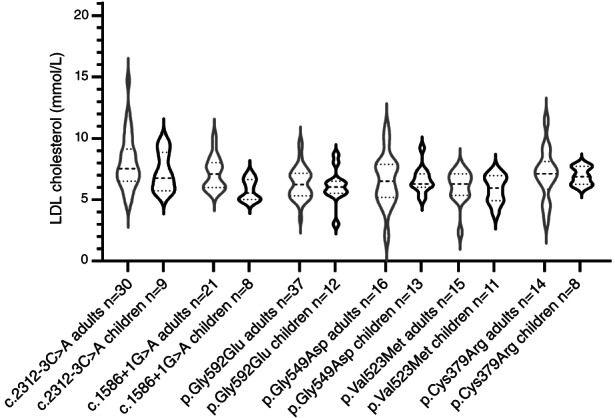
Violin plots reporting the range of low‐density lipoprotein (LDL)‐cholesterol levels observed among patients with the six most frequent pathogenic variants. Gray and black violins represent the adult and pediatric patients, respectively. The violin shape represents the smoothed frequency distribution of the LDL‐cholesterol values expressed in mmol/L. The continuous horizontal line within each value represents the distribution median, whereas the dashed lines represent the first and the third quartile of value distribution in each group

In order to evaluate the variability of LDL‐c levels among patients with the same variant, we analyzed the unrelated heterozygous patient with the 6 most‐frequent variants. The violin plots in Figure 4 highlight that the variant c.2312‐3C>A is associated with the widest range of LDL‐c values in adults (10.13 mmol/L of difference between maximum and minimum values observed among 30 unrelated patients). The lowest variability was observed for the c.1586+1G>A that showed a range of 4.79 mmol/L in adults and 2.17 mmol/L in children. For all variants, the variability of LDL‐c levels is lower in children than in adults. As expected, for all variants, the LDL‐c value ranges are lower in children than in adults.

### 3.3 Analysis of DLCN and Simon Broome criteria

**FIGURE 5:**
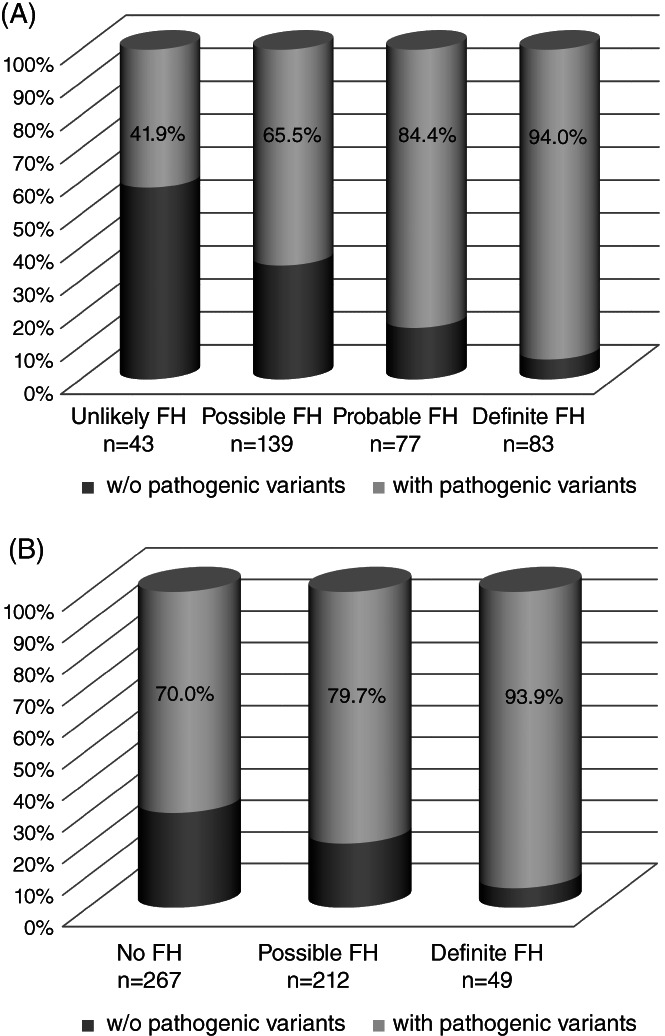
Frequency of patients with pathogenic variants in groups based on clinical diagnosis according to Dutch Lipid Clinic Network and Simon Broome criteria. The cumulative histograms represent the frequency of the patients with and without pathogenic variants diagnosed according to clinical criteria for FH diagnosis. (A) Dutch Lipid Clinic Network criteria applied only on adult patients. (B) Simon Broome Criteria applied on all patients

We calculated the DLCN score for adult patients and performed the clinical diagnosis accordingly. As reported in Figure 5A, the most of adult patients are classified as possible FH and there are 43 patients for which the calculated score should have excluded the diagnosis. As expected, the frequency of pathogenic variants gradually increased from Unlikely FH to Definite FH, highlighting that the higher scores were more suggestive of a variant presence respect to the lower ones. However, it should be noted that among patients with Unlikely FH, 41.9% carried a pathogenic variant. On the other hand, the frequency of pathogenic variants among Definite FH patients was very high (94%) but not the 100%.

As DLCN criteria cannot be applied to pediatric patients, clinical diagnosis according to Simon Broome criteria, that are valid for both adults and children, was also analyzed. One half of patients did not fulfill Simon Broome criteria and were classified as No FH (267/528), whereas only 49 were classified as Definite FH (Figure 5B). Also in this case, the prevalence of a causative variant increase from No FH to Definite FH. Surprisingly, the frequency of No FH patients carrying a pathogenic variant was very high (70%), whereas the frequency of causative variants among Definite FH was similar to that observed using DLCN criteria (about 94%—Figure 5B).

## 4 DISCUSSION

In this study, we reported the results of a retrospective analysis of 528 unrelated FH patients undergoing genetic screening and recruited at different Italian centers, mainly from the south of the country. After the pathogenicity evaluation, we identified 107 pathogenic variants and 36 VUS. It should be noted that some VUS could have a pathogenic role, but the evidences now available do not allow to correctly classify these as pathogenic or benign and then should be further investigated by functional studies as well as by additional screening of FH patients, perhaps including segregation studies. In particular, most of *APOB* variants were classified as VUS according to a recent ClinVar data revision.^23^ Whereas for PCSK9 variants, we were able to better classify as pathogenic several variants thank to a previous extensive functional characterization.^24^, ^25^, ^26^

We analyzed the lipid profile of patients with different genetic status demonstrating that HeFH patients with a null variant showed a worse phenotype than those with a defective variant. This result can be explained by the lower residual LDLR function associated with null variants than in that associated with the defective ones.^27^ In addition, both groups of HeFH patients, with defective and null variants, showed higher basal LDL‐c levels than patients with a clinical suspicion of FH without pathogenic variants. Furthermore, despite the low number of patients with a missense variant in *APOB* or *PCSK9* genes (*n* = 7), we were able to observe lower values of LDL‐c and LDL/HDL ratio in these patients compared with patients with a missense variant in *LDLR* gene. This result underline that variants in *LDLR* gene are associated with a worse phenotype as reported in few other studies.^10^, ^28^ Only one study reported a worse phenotype for carriers of PCSK9 pathogenic variants.^29^

The variant types associated with different phenotypes can provide also a prognostic evaluation improving the patient management, in particular in children that can benefit for a prompt intervention.^30^ Also Khera et al. reported an association between null (loss of function) variants and a worse phenotype respect to missense pathogenic variants.^31^

The genetic spectrum of FH emerging from this study highlights the three main characteristics of FH genetics, namely, the genetic heterogeneity, the presence of variant clusters and the phenotypic variability.^4^ In facts, we identified 99 different causative variants, with six variants in the *LDLR* gene accounting for more than one half of patients. Three of these variants [p.(Gly592Glu), p.(Gly549Asp) and p.(Val523Met)] have already been identified as variant clusters in Italy and for the p.(Gly549Asp) a founder effect was verified hypothesizing its importation from Greece.^32^ For the other high‐frequency variants, a founder effect could be hypothesized although it was not yet demonstrated.

As to phenotypic variability, we performed the analysis on unrelated patients carrying the same variant at heterozygous status considering the 6 variants for which we dispose of a large patient number. A large variability was observed with range of values reaching 10.13 mmol/L of range in adults. Level ranges are smaller in children than in adults, suggesting that the age and the lifestyle had a strong impact on determining the variability associated with a variant presence.

Despite the use of not stringent criteria for patient inclusion, we identified pathogenic variants in 76.1% of patients. Stratifying for DLCN score we found increasingly percentage of genetically confirmed FH from unlikely to definite FH diagnosis, as also observed in an expanded Italian study.^33^ The presence of a pathogenic variant in more than 40% of adult patients with unlikely FH according to DLCN criteria, suggest that in order to identify all FH patients, less stringent criteria should be used. Of course, the analysis of a high patient number deriving from less stringent criteria would lead to high costs for genetic screening, but the identification of all FH patients and of their causative variant would lead to a really effective cascade screening.

Applying Simon Broome criteria, the most of patients resulted as No FH, probably due to the high LDL‐c levels used as inclusion threshold as well as to some missing data. For children, the European Atherosclerosis Society suggested to decrease the LDL‐c threshold to 130 mg/dl (3.36 mmol/L) in case of a parent with a genetic diagnosis.^34^ Unfortunately, in the most of countries the FH remain mainly genetically undiagnosed and consequently, this threshold could be applied only to few children. These data underline the urge for new clinical criteria applicable on children. In the meantime, the creation of national registries will improve the identification of FH patients, greatly improving the cascade screening.^35^, ^36^

According to both DLCN and Simon Broome criteria, among Definite FH the frequency of pathogenic variants was very high but did not reach the totality of patients. These data suggest that other genetic causes of hypercholesterolemia could be present such as the FH phenocopies,^4^ that would be identified by extended NGS panel. The decreasing costs of NGS make this method the most cost‐effective for FH diagnosis, also improving the detection of double heterozygous patients. Unfortunately, the greater is the amount of data the more is complicated the analysis, in particular regarding the pathogenicity assessment. The identification of two or more variants in a patient could be mis‐interpreted as a double heterozygosis as one of our patients with a *LDLR* and a *PCSK9* variant, which variants were recently characterized verifying that only the latter variant was functional.^24^ As to HoFH, here we report two additional HoFH cases respect to our very recent study and, based on the number of HoFH identified, we established that the frequency of this rare FH form is at least 1:286000 in the Campania region, even higher than recently described. This finding further highlights the need for genetic screening of hypercholesterolemic patients in order to identify all the FH patients and apply the cascade screening for cardiovascular prevention.

Many HoFH patients in this study did not reach the LDL‐c cut‐off proposed by EAS for clinical diagnosis of HoFH (13 mmoL/L for untreated and 8 mmol/L for treated patients).^37^ However, EAS guidelines also underline that these values could be lower. On the other hand, it should be noted the opposite situation: patients with very high LDL‐c levels that were confirmed as HeFH even at NGS analysis. This aspect highlights the large overlap of LDL‐c levels between HeFH and HoFH and the need for an accurate genetic screening allowing to confirm the patient genotype.

For NGS analysis we used a small panel including all known causative genes; this kind of panel is very useful for routine diagnosis because keeps the cost low and can be easily performed and analyzed. On the other hand, the extended panels, including genes leading to FH phenocopies, would identify a high number of rare variants in other lipid‐associated genes. Unfortunately, to date the pathogenicity evidences of variants in FH‐phenocopy genes are too few to easily establish their role in FH development.^38^

An additional confounding factor in FH patient identification is represented by the partial overlap with familial combined hyperlipidemia (MIM: 602491), characterized also by the presence of hypertriglyceridemia. In fact, several studies reported the presence of FH‐causative variants among patients with a diagnosis of familial combined hyperlipidemia^39^, ^40^, ^41^ leading to a recently proposed clinical classification of an additional FH form with hypertriglyceridemia. The frequency of hypertriglyceridemia among FH patients is slightly below 20%, sensibly lower than in patients with familial combined hyperlipidemia.^42^, ^43^ By the ROC curve analysis, we identified the potential cut‐off points to discriminate the presence of a pathogenic variant in adults and children. As these cut‐off points are a good compromise between sensitivity and specificity, they are high LDL‐c values, whereas the LDL‐c levels associated with the identification of all patients (100% sensitivity) are very low, namely, 2.87 mmol/L in children and 2.07 mmol/L in adults. Of course, these values cannot be used in clinical practice to select FH patients because they would include a large number of non‐genetic hypercholesterolemia. However, this result highlights that a diagnosis of FH should not be excluded in case of low LDL‐c values, if a dominant transmission of the hypercholesterolemia is observed within the family.

The ROC curves analyses showed higher AUC in children than in adults, suggesting that high LDL‐c levels are more suggestive of FH in children than in adults, in which the high cholesterol levels can be naturally observed independently from the presence of a pathogenic variant. Another evidence of this phenomenon can be inferred from the lower LDL‐c variability observed in children sharing the same pathogenic variants respect to the adults with the same variants. The ROC curves of non‐HDL cholesterol and LDL‐c are similar, suggesting that the use of non‐HDL cholesterol could be considered as an alternative parameter for patient identification. To date, the use of non‐HDL‐cholesterol is not recommended for dyslipidemia characterization as recently reported in a recent consensus paper^44^ but additional studies would better clarify the usefulness of this parameter. The advantage of non‐HDL‐cholesterol is that it can be calculated also on non‐fasting samples representing a good opportunity or lipid screening in children.^45^

In conclusion, our data suggest that LDL‐c levels lower than thresholds proposed by existing criteria cannot be always considered an exclusion criterium because an extreme variability is often associated with a single variant. The evaluation of a dominant transmission of the hypercholesterolemic trait would improve FH patient identification. A correct genetic diagnosis, including an accurate pathogenicity evaluation, would improve the patient prognosis because the different variant types and genetic status are associated with different phenotypes.

## CONFLICT OF INTEREST

The authors declare no conflict of interest.

## Supporting information


**FIGURE S1** **Frequency of the different pathogenic variants identified in unrelated HeFH patients.** Each slice of the donut graph represents a different variant according to the legend. The frequency of the different variant type is indicated by the external lines, whereas the portion of patients with the 6 most frequent variants is indicated by the internal line.
**FIGURE S2. Position and frequency of the six most frequent pathogenic variants identified in unrelated HeFH patients.** Scheme of LDLR gene with blue boxes representing the exons. The position of the six most frequent pathogenic variants together with the number and the percentage of heterozygous familial hypercholesterolemia patients in which they were identified is reported.
**FIGURE S3. Violin plot representing LDL‐cholesterol values observed in pediatric and adult patients with different genetic statuses.** Gray and black violins represent the pediatric and adult patients, respectively. The violin shape represents the smoothed frequency distribution of the LDL‐cholesterol values expressed in mmol/L. The continuous horizontal line within each value represents the distribution median, whereas the dashed lines represent the first and the third quartile of value distribution in each group. Statistical significances corrected for multiple comparisons obtained by Dunn's test are reported. V‐: patients without pathogenic variants; Def HeFH: heterozygous patients with defective variants; Null HeFH: heterozygous patients with null variants; HoFH: homozygous patients.
**FIGURE S4. ROC curves evaluating the ability of lipid parameters to distinguish between patients with and without pathogenic variants.** The ROC curve is indicated with bold line and open circles represent the best criterion points. Light line indicates the 95% confidence interval (CI). Dashed line indicates the bisector. AUC, area under the curve.

**TABLE S1** Genetic status of unrelated Homozygous patients
**TABLE S2**. Variants of uncertain significance identified during genetic screening
**TABLE S3**. Demographic, biochemical and clinical features of adult and pediatric FH patients

## Data Availability

Data are available from the corresponding author upon reasonable request.
