# Supplementary material for: Genetic spectrum of familial hypercholesterolemia and correlations with clinical expression: Implications for diagnosis improvement
Source: Clin Genet. 2021 Aug 3;100(5):529–41. doi: 10.1111/cge.14036 (PMC9291778; doi:10.1111/cge.14036)
Supplement: Supplementary file 1 — FIGURE S1 Frequency of the different pathogenic variants identified in unrelated HeFH patients. Each slice of the donut graph represents a different variant according to the legend. The frequency of the different variant type is indicated by the external lines, whereas the portion of patients with the 6 most frequent variants is indicated by the internal line. FIGURE S2. Position and frequency of the six most frequent pathogenic variants identified in unrelated HeFH patients. Scheme of LDLR gene with blue boxes representing the exons. The position of the six most frequent pathogenic variants together with the number and the percentage of heterozygous familial hypercholesterolemia patients in which they were identified is reported. FIGURE S3. Violin plot representing LDL‐cholesterol values observed in pediatric and adult patients with different genetic statuses. Gray and black violins represent the pediatric and adult patients, respectively. The violin shape represents the smoothed frequency distribution of the LDL‐cholesterol values expressed in mmol/L. The continuous horizontal line within each value represents the distribution median, whereas the dashed lines represent the first and the third quartile of value distribution in each group. Statistical significances corrected for multiple comparisons obtained by Dunn's test are reported. V‐: patients without pathogenic variants; Def HeFH: heterozygous patients with defective variants; Null HeFH: heterozygous patients with null variants; HoFH: homozygous patients. FIGURE S4. ROC curves evaluating the ability of lipid parameters to distinguish between patients with and without pathogenic variants. The ROC curve is indicated with bold line and open circles represent the best criterion points. Light line indicates the 95% confidence interval (CI). Dashed line indicates the bisector. AUC, area under the curve. [file CGE-100-529-s002.docx]

**Supplemental figures**


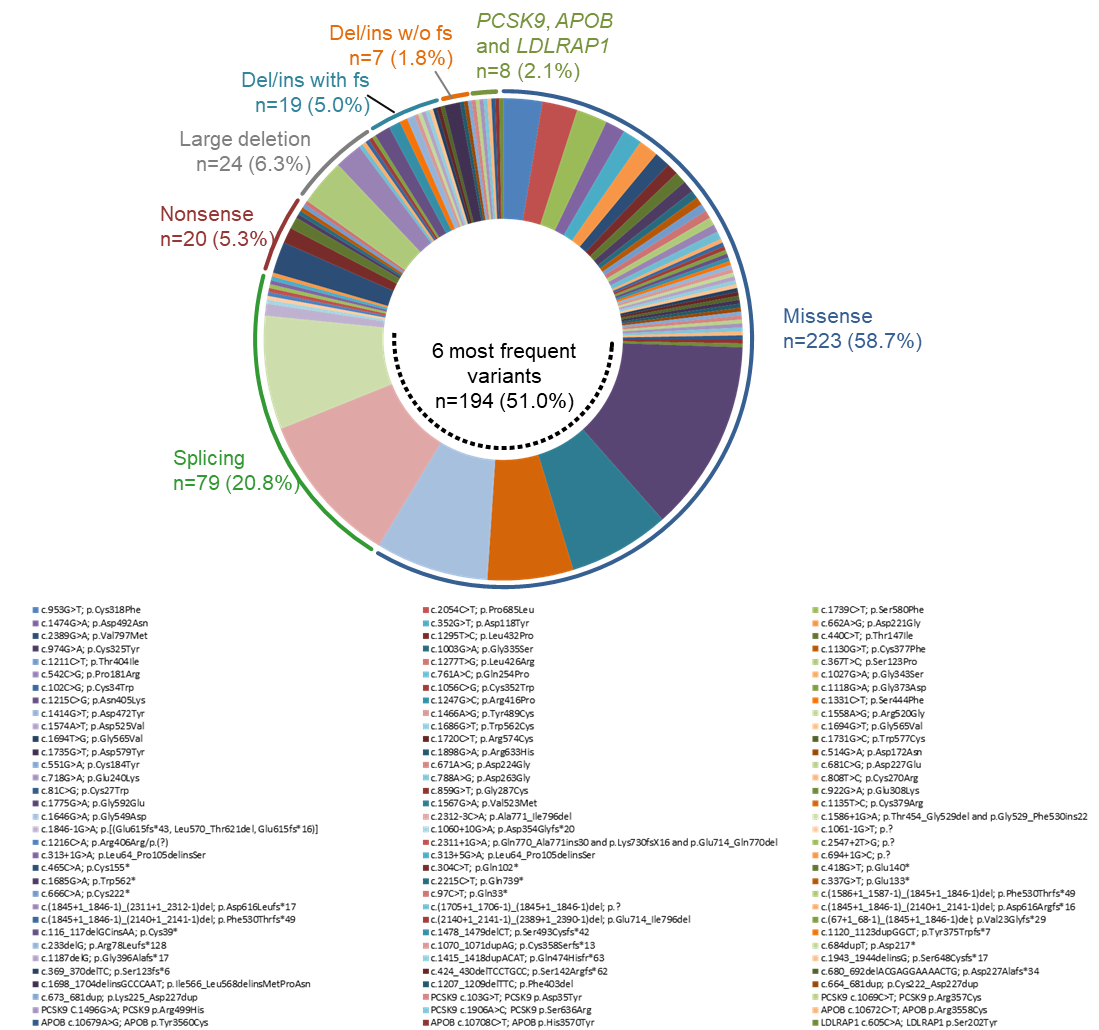


**Supplemental Figure 1. Frequency of the different pathogenic variants identified in unrelated HeFH patients.** Each slice of the donut graph represents a different variant according to the legend. The frequency of the different variant type is indicated by the external lines, whereas the portion of patients with the 6 most frequent variants is indicated by the internal line.


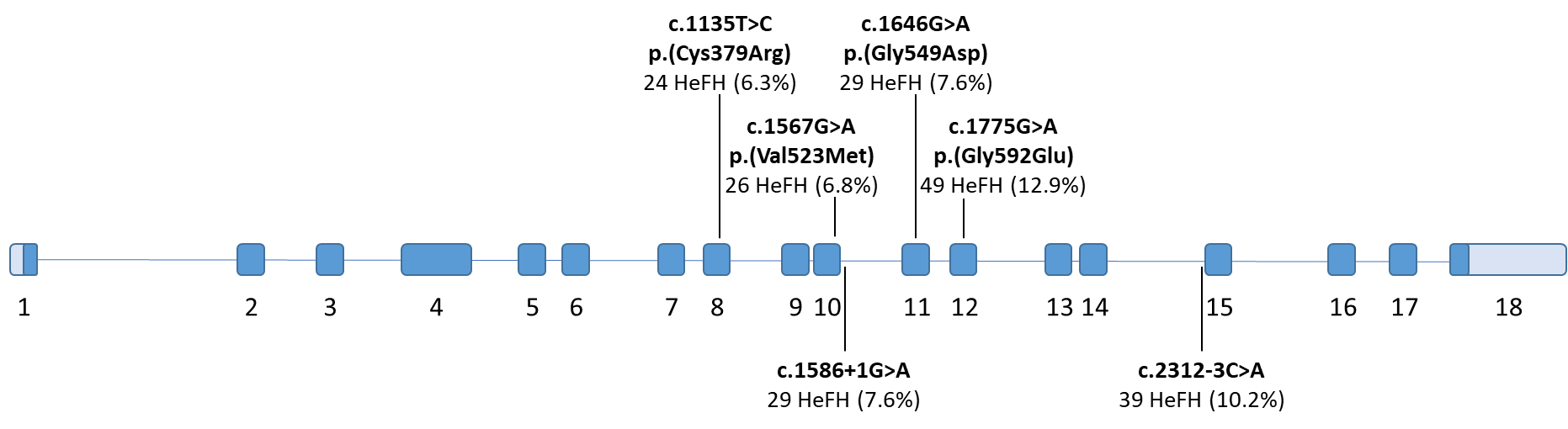


**Supplemental Figure 2. Position and frequency of the six most frequent pathogenic variants identified in unrelated HeFH patients.** Scheme of LDLR gene with blue boxes representing the exons. The position of the six most frequent pathogenic variants together with the number and the percentage of heterozygous familial hypercholesterolemia patients in which they were identified is reported.


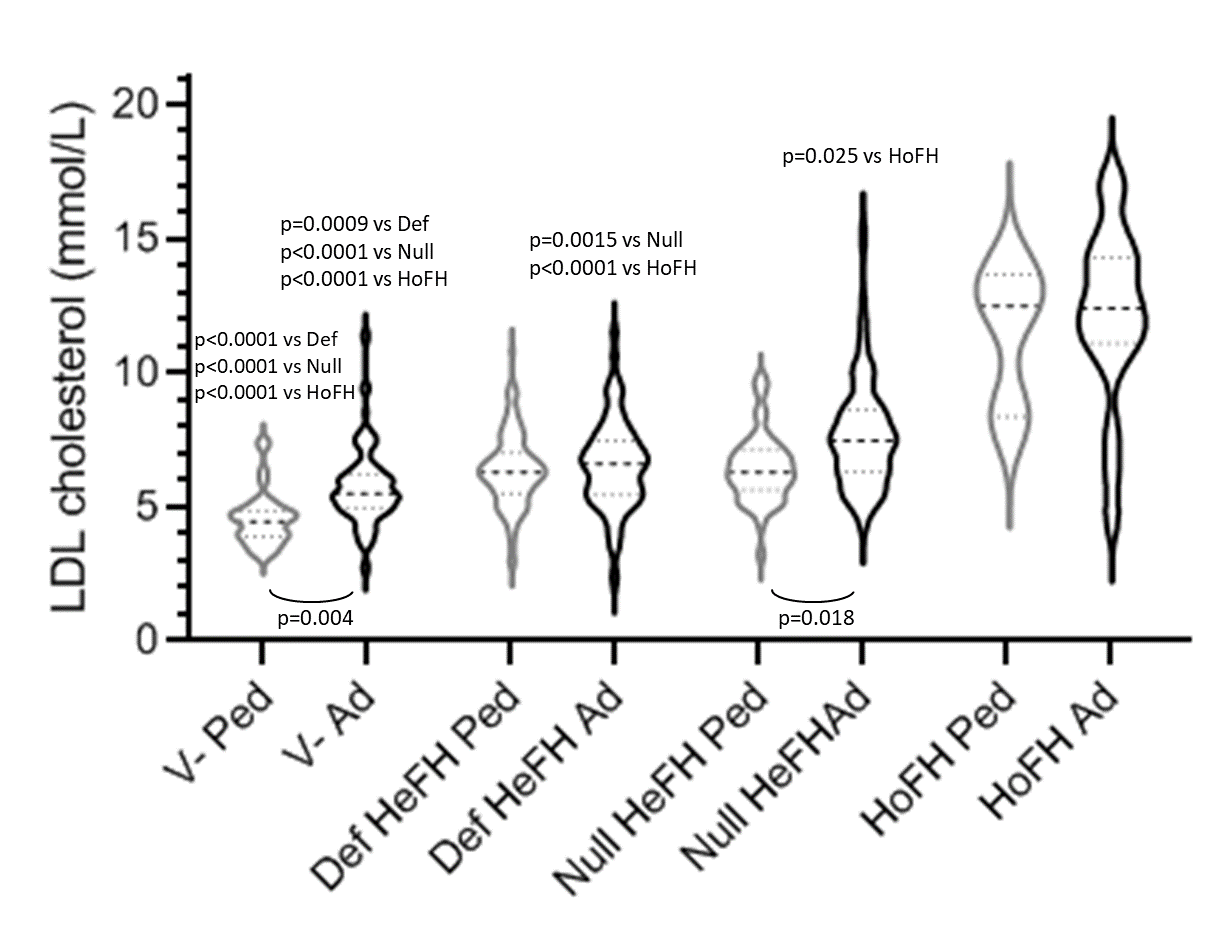


**Supplemental Figure 3. Violin plot representing LDL-cholesterol values observed in pediatric and adult patients with different genetic statuses.** Grey and black violins represent the pediatric and adult patients, respectively. The violin shape represents the smoothed frequency distribution of the LDL-cholesterol values expressed in mmol/L. The continuous horizontal line within each value represents the distribution median, whereas the dashed lines represent the first and the third quartile of value distribution in each group. Statistical significances corrected for multiple comparisons obtained by Dunn’s test are reported. V-: patients without pathogenic variants; Def HeFH: heterozygous patients with defective variants; Null HeFH: heterozygous patients with null variants; HoFH: homozygous patients.

**
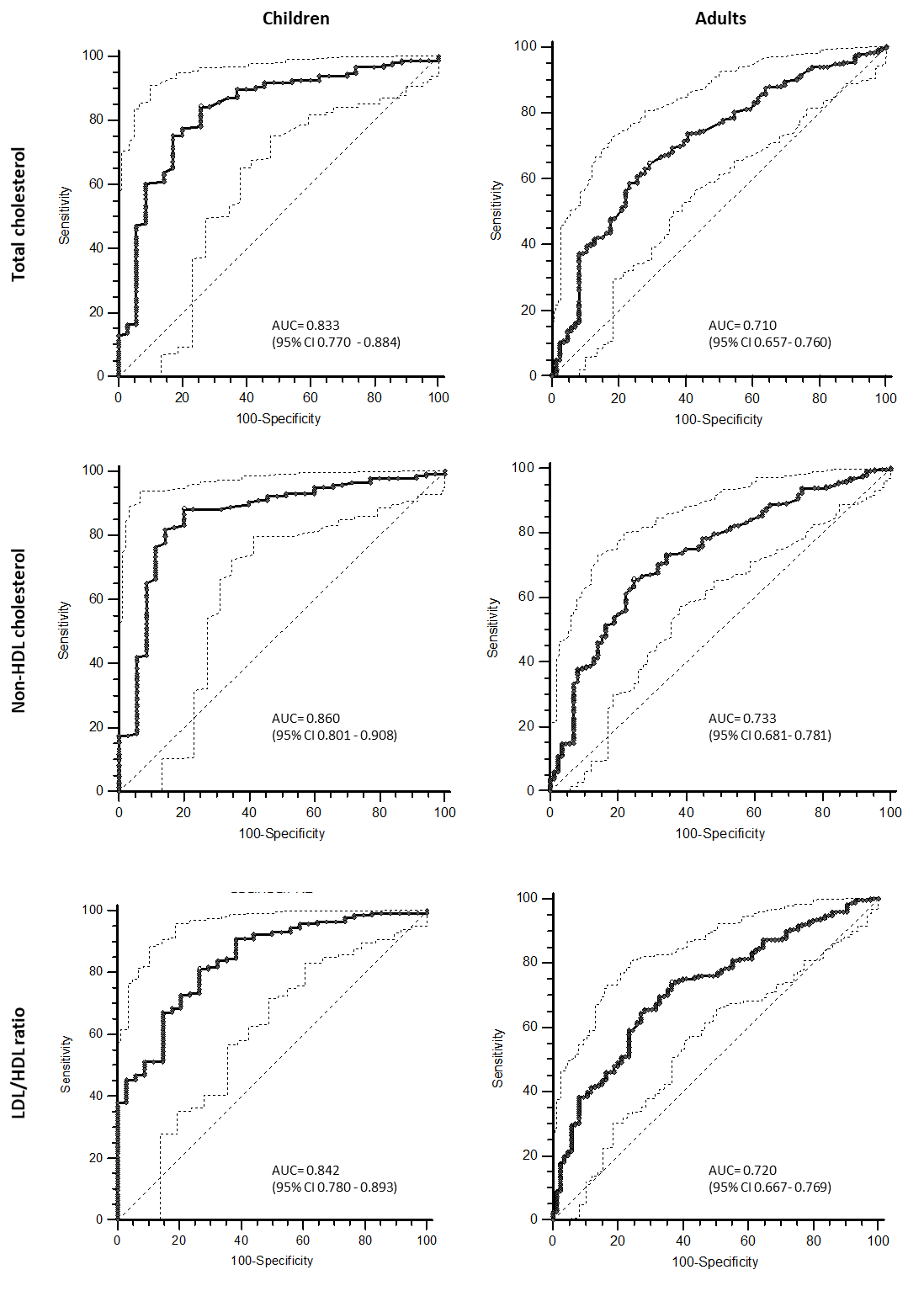
**

**Supplemental Figure 4. ROC curves evaluating the ability of lipid parameters to distinguish between patients with and without pathogenic variants.** The ROC curve is indicated with bold line and open circles represent the best criterion points. Light line indicates the 95% confidence interval (CI). Dashed line indicates the bisector. AUC, area under the curve.
