# Supplementary material for: Genetic spectrum of familial hypercholesterolemia and correlations with clinical expression: Implications for diagnosis improvement
Source: Clin Genet. 2021 Aug 3;100(5):529–41. doi: 10.1111/cge.14036 (PMC9291778; doi:10.1111/cge.14036)
Supplement: Supplementary file 2 — TABLE S1 Genetic status of unrelated Homozygous patients TABLE S2. Variants of uncertain significance identified during genetic screening TABLE S3. Demographic, biochemical and clinical features of adult and pediatric FH patients [file CGE-100-529-s001.docx]

**Supplemental Table 1. Genetic status of unrelated Homozygous patients**

| **Patient ID** | **Age (years)** | **Gender** | **Nucleotide** | **Protein** |
| --- | --- | --- | --- | --- |
| HoFH-1 | 29 | male | LDLR:c.[367T>C];[1478_1479delCT] | LDLR:p.[(Ser123Pro)];[(Ser493Cysfs*42)] |
| HoFH-2 | 7 | female | LDLR:c.[407A>T];[1775G>A] | LDLR:p.[(Asp136Val)];[(p.Gly592Glu)] |
| HoFH-3 | 22 | male | LDLR:c.[1775G>A];[1775G>A] | LDLR:p.[(Gly592Glu)];[(Gly592Glu)] |
| HoFH-4 | 33 | female | LDLR:c.[1646G>A];[1739C>T] | LDLR:p.[(Gly549Asp)];[(Ser580Phe)] |
| HoFH-5 | 64 | female | LDLR:c.[1775G>A];[2054C>T] | LDLR:p.[(Gly592Glu)];[(Pro685Leu)] |
| HoFH-6 | 35 | male | LDLR:c.[1135T>C];[1135T>C] | LDLR:p.[(Cys379Arg)];[(Cys379Arg)] |
| HoFH-7 | 62 | female | LDLR:c.[1135T>C];[1586+5G>A] | LDLR:p.[(Cys379Arg)];[(Thr454_Gly529del)] |
| HoFH-8 | 46 | male | LDLR:c.[1-156C>T];[1-156C>T] | LDLR:p.[(?)];[(?)] |
| HoFH-9 | 10 | male | LDLR:c.[1135T>C];[1775G>A] | LDLR:p.[(Cys379Arg)];[(Gly592Glu)] |
| HoFH-10 | 53 | male | LDLR:c.[1130G>T];[2476C>A] | LDLR:p.[(Cys377Phe)];[(Pro826Thr)] |
| HoFH-11 | 40 | female | LDLR:c.[1567G>A];[2054C>T] | LDLR:p.[(Val523Met)];[(Pro685Leu)] |
| HoFH-12 | 49 | female | LDLR:c.[323C>T];[1586+1G>A] | LDLR:p.[(Thr108Met)];[(Thr454_Gly529del),(Gly529_Phe530ins22)] |
| HoFH-13 | 9 | male | LDLR:c.[1739C>T];[1775G>A] | LDLR:p.[(Ser580Phe)];[(Gly592Glu)] |
| HoFH-14 | 46 | male | LDLR:c.[304C>T];[718G>A] | LDLR:p.[(Gln102*)];[(Glu240Lys)] |
| HoFH-15 | 36 | male | LDLR:c.[352G>T];[1646G>A] | LDLR:p.[(Asp118Tyr)];[(Gly549Asp)] |
| HoFH-16 | 55 | female | LDLR:c.[974G>A];[(940+1_941-1)_(2311+1_2312-1)dup] | LDLR:p.[(Cys325Tyr)];[(Gly314_Gln770dup)] |
| HoFH-17 | 63 | male | LDLR:c.[463T>C];[1567G>A] | LDLR:p.[(Cys155Arg)];[(Val523Met)] |
| HoFH-18 | 28 | female | LDLR:c.[1775G>A];[2054C>T] | LDLR:p.[(Gly592Glu)];[(Pro685Leu)] |
| HoFH-19 | 48 | female | LDLR:c.[727T>C];[1775G>A] | LDLR:p.[(Cys243Arg)];[(Gly592Glu)] |
| HoFH-20 | 29 | female | LDLR:c.[1135T>C];[1567G>A] | LDLR:p.[(Cys379Arg)];[(Val523Met)] |
| HoFH-21 | 68 | female | LDLR:c.(1586+1_1587-1)_(*450_?)del PCSK9c.1906A>C | LDLR:p.(?) PCSK9p.(Ser636Arg) |
| HoFH-22 | 35 | female | LDLR:c.[1775G>A];[1775G>A] | LDLR:p.[(Gly592Glu)];[(Gly592Glu)] |

**Supplemental Table 2. Variants of uncertain significance identified during genetic screening**

| **Gene** | **Nucleotide** | **Protein** | **Variant ID** | **MAF in GnomAD** | **HGMD** |
| --- | --- | --- | --- | --- | --- |
| *LDLR* | LRG_274t1:c.-19C>T | p.(?) | n.a. | n.a. | n.r. |
| *LDLR* | LRG_274t1:c.58G>A | p.(Gly20Arg) | rs147509697 | A=0.0007306 | DM? CM022807 |
| *LDLR* | LRG_274t1:c.79T>A | p.(Cys27Ser) | n.a. | n.a. | n.r. |
| *LDLR* | LRG_274t1:c.303G>A | p.(Glu101=) | rs1600705245 | n.a. | n.r. |
| *LDLR* | LRG_274t1:c.829G>A | p.(Glu277Lys) | rs148698650 | A=0.0005056 | DM? CM950757 |
| *LDLR* | LRG_274t1:c.892A>G | p.(Met298Val) | rs730882092 | G=0.00001193 | DM?  CM176144 |
| *LDLR* | LRG_274t1:c.1060+40G>A | p.(?) | rs192390193 | A=0.002458 | n.r. |
| *LDLR* | LRG_274t1:1060+156C>A | p.(?) | n.a. | n.a. | n.r. |
| *LDLR* | LRG_274t1:c.1061-143C>T | p.(?) | rs78598082 | n.a. | n.r. |
| *LDLR* | LRG_274t1:c.1061-112G>C | p.(?) | n.a. | n.a. | n.r. |
| *LDLR* | LRG_274t1:c.1061-8T>C | p.(?) | rs72658861 | C=0.005498 | DM?  CS961610 |
| *LDLR* | LRG_274t1:c.1336C>G | p.(Leu446Val) | rs375651668 | G=0.000003980 | n.r. |
| *LDLR* | LRG_274t1:c.1510A>G | p.(Lys504Glu) | rs730882103 | G=0.00001193 | DM  CM094970 |
| *LDLR* | LRG_274t1:c.1706-10G>A | p.(?) | rs17248882 | A=0.002220 | DM?  CS971794 |
| *LDLR* | LRG_274t1:c.1186+31G>A | p.(?) | rs572365788 | A= 0.000008193 | n.r. |
| *LDLR* | LRG_274t1:c.1836C>T | p.(Ala612=) | rs143872778 | T=0.0001874 | DM  CM078328 |
| *LDLR* | LRG_274t1:c.1988-115C>A | p.(?) | n.a. | n.a. | n.r. |
| *LDLR* | LRG_274t1:c.2101G>A | p.(Gly701Ser) | rs368838866 | A=0.00008853 | DM CM055380 |
| *LDLR* | LRG_274t1:c.2140+5G>A | p.(?) | rs72658867 | A=0.007223 | DFP  CS991432 |
| *LDLR* | LRG_274t1:c.2177C>T | (Thr726Ile) | rs45508991 | T=0.005488 | DM?  CM920469 |
| *APOB* | NM_000384.3:c.9803C>T | p.(Ser3268Leu) | rs755753053 | A=0.00004778 | n.r. |
| *APOB* | NM_000384.3:c.9835A>G | p.(Ser3279Gly) | rs12720854 | C=0.004576 | DM? |
| *APOB* | NM_000384.3:c.9916G>A | p.(Val3306Ile) | ? | n.a. | n.r. |
| *APOB* | NM_000384.3:c.10131G>A | p.(Leu3377=) | rs1799812 | T=0.005862 | n.r. |
| *APOB* | NM_000384.3:c.10819C>T | p.(Pro3607Ser) | ? | n.a. | n.r. |
| *APOB* | NM_000384.3:c.10897T>C | p.(Trp3633Arg) | ? | n.a. | n.r. |
| *APOB* | NM_000384.3:c.11087T>C | p.(Ile3696Thr) | rs370096275 | G=0.000007082 | n.r. |
| *APOB* | NM_000384.3:c.11354C>T | p.(Thr3785Ile) | rs143710616 | A=0.0004744 | n.r. |
| *APOB* | NM_000384.3:c.11761G>A | p.(Val3921Ile) | rs72654409 | T=0.0009091 | DM? |
| *APOB* | NM_000384.3:c.12536C>G | p.(Thr4179Ser) | ? | n.a. | DM |
| *APOB* | NM_000384.3:c.12940A>G | p.(Ile4314Val) | rs72654423 | 0.005308 | n.r. |
| *PCSK9* | LRG_275t1:c.720C>T | p.(Gly240=) | rs41297883 | T=0.004983 | n.r. |
| *PCSK9* | LRG_275t1:c.991C>G | p.(Pro331Ala) | ? | n.a. | n.r. |
| *PCSK9* | LRG_275t1:c.1394C>T | p.(Ser465Leu) | rs778849441 | T=0.00002387 | DM CM1514511 |
| *PCSK9* | LRG_275t1:c.1405C>T | p.(Arg469Trp) | rs141502002 | T=0.0008878 | DM  CM054796 |
| *PCSK9* | LRG_275t1:c.1928A>G | p.(His643Arg) | ? | n.a. | n.r. |

**Supplemental Table 3. Demographic, biochemical and clinical features of adult and pediatric FH patients**

|  | **Adult FH patients n=342** | **Pediatric FH patients n=186** | **Statistical significant difference** |
| --- | --- | --- | --- |
| Age (years) | 42 (27-54) | 10 (7-13) | p<0.0001 |
| Sex *n males* (%) | 147 (43.0%) | 83 (44.6%) | ns |
| Body Mass Index (kg/m^2^) | 24.9 (22.4-27.7) | 17.3 (15.7-20.7) | p<0.0001 |
| Smokers *n* (%) | 92 (26.9%) | 2 (1.1%) | p=0.027 |
| Total cholesterol (mmol/L) | 8.50 (7.53-9.90) | 7.78 (6.69-8.74) | p<0.0001 |
| HDL-cholesterol (mmol/L) | 1.30 (1.12-1.58) | 1.31 (1.14-1.61) | ns |
| LDL-cholesterol (mmol/L) | 6.57 (5.44-7.82) | 6.07 (4.99-6.97) | p<0.0001 |
| Non-HDL cholesterol | 7.12 (6.01-8.55) | 6.40 (5.32-7.24) | p<0.0001 |
| LDL/HDL ratio | 5.00 (3.73-6.60) | 4.43 (3.40-5.52) | p=0.001 |
| Triglycerides (mmol/L) | 1.13 (0.94-1.65) | 0.89 (0.71-1.13) | p<0.0001 |
| Tendon xanthomas *n* (%) | 42 (12.3%) | 1 (0.5%) | p<0.0001 |
| Corneal arcus *n* (%) | 44 (12.9%) | 1 (0.5%) | p<0.0001 |
| Premature CHD *n* (%) | 43 (12.6%) | 0 | p=0.040 |
| Carotid plaque *n* (%) | 59 (17.2%) | 0 | p=0.028 |

FH, familial hypercholesterolemia; CHD, Coronary heart disease
